# Determination of reactive oxygen species in mainstream smoke from various heated tobacco products

**DOI:** 10.1265/ehpm.25-00121

**Published:** 2025-08-29

**Authors:** Shoichi Nishimoto-Kusunose, Yohei Inaba, Kanae Bekki, Akira Ushiyama

**Affiliations:** Department of Environmental Health, National Institute of Public Health, 2-3-6 Minami, Wako-shi, Saitama 351-0197, Japan

**Keywords:** Tobacco, Heated tobacco products, Oxidative stress, Reactive oxygen species

## Abstract

**Background:**

Although smoking rates have been declining worldwide, new types of tobacco products have been gradually spreading in recent years, especially in Japan, where heated tobacco products (HTPs) users are rapidly increasing. Oxidative stress caused by reactive oxygen species (ROS) is one of the causes of smoking-induced carcinogenesis, respiratory diseases, and cardiovascular diseases. However, information on the amount of ROS contained in mainstream smoke from HTPs is limited. In this study, we measured the amount of ROS generated from HTPs to evaluate the oxidative stress-related toxicity of HTPs.

**Methods:**

IQOS ILUMA, glo hyper+, and Ploom X ADVANCED were used as the HTP devices. Mainstream smoke was collected from each HTP according to Health Canada Intense regime (smoke volume, 55 mL; smoke duration, 2 s). The collected ROS were reacted with 2,7′-dichlorodihydrofluorescein reagents, and the amount of ROS was calculated as H_2_O_2_ equivalent from the fluorescence intensity obtained.

**Results:**

The ROS in the mainstream smoke from IQOS ILUMA, glo hyper+ (high-temperature mode), and Ploom X ADVANCED was found to be 48.8 ± 8.6, 86.6 ± 12.6, and 40.8 ± 5.7 nmol H_2_O_2_/stick, respectively (*n* = 6, mean ± standard deviation), with the highest being from glo hyper+ (high-temperature mode). The amount of ROS was significantly higher in the high-temperature mode of glo hyper+ than in the standard mode of glo hyper+. Additionally, the estimated amount of ROS from smoking 20 heated sticks per day (674–2160 nmol H_2_O_2_/day) was equivalent to 2.2–96 times the amount of daily exposure to ROS in the urban atmosphere (approximately 22–300 nmol H_2_O_2_/day).

**Conclusions:**

We found that ROS is generated from HTPs of different devices. This study suggests that HTPs users may be exposed to much more ROS than they are exposed to in normal life.

## Background

It has long been recognized that tobacco smoking poses a serious risk to human health because it can lead to cancer, cardiovascular disease, and respiratory disease [1–3]. Smoking rates are gradually declining worldwide because of the increased awareness of the negative health impacts of tobacco [4]. However, new types of tobacco, such as electronic cigarettes and heated tobacco products (HTPs), have emerged in recent years. Particularly in Japan, following the launch of the IQOS in 2014, HTPs have spread rapidly, surpassing the use rate of conventional cigarettes among those in their 20s and 30s, according to a 2024 survey by the Ministry of Health, Labor and Welfare. The high-temperature HTPs, which directly heats tobacco leaves at 200–350 °C, are gaining popularity among HTPs [5].

Although HTPs manufacturers advertise that HTPs contain fewer harmful substances than conventional cigarettes because they do not involve combustion, it is reported that no considerable difference is noted between HTPs and conventional cigarettes with respect to the total compounds in mainstream smoke, and some ingredients are even more abundant in HTPs than in conventional cigarettes [6, 7]. Additionally, it has been found that the amount of toxic substances and health effects are not necessarily linked, and individuals who smoke cigarettes, even in small amounts, are at significantly increased risk of cardiovascular disease compared to nonsmokers [8].

Reactive oxygen species (ROS) are known to be one of the causes of tobacco-induced carcinogenesis, respiratory diseases, and cardiovascular diseases [2, 9, 10]. ROS is a general term for oxygen species such as hydrogen peroxide, hydroxyl radicals, ROO radicals, and organic peroxides, and they are highly reactive and cause oxidative damage to biological and other components [11]. Their exposure causes various adverse effects on living organisms, such as DNA damage, structural protein damage, and enzyme activity reduction; thus, they are considered a group of toxic substances whose actual status needs to be understood [12]. Many studies have shown that conventional cigarettes produce large amounts of ROS [13, 14]. Since HTPs also contain various harmful substances, it is likely that they produce similar types of ROS. Furthermore, as the number of HTP users and their sales continue to grow rapidly, it is likely that their use will spread across a wider range of age groups [15]. This means that more people may be continuously exposed to the harmful effects of ROS, potentially increasing the risk of diseases such as cancer, cardiovascular disease, and respiratory disease. To prevent the users from unknowingly suffering health effects caused by ROS, it is urgently necessary to quantify the ROS generated from HTPs and to assess the associated health risks.

One of the most common methods used to measure total ROS is a chemical assay employing the fluorescent reagents, such as 2′,7′-dichlorodihydrofluorescein (DCFH) [13, 14]. Reports on the direct analysis of ROS generated from HTPs are inadequate because only one case has been reported [16]. It should be noted that this report employed DCFH to measure the amount of ROS, but the method was not sufficiently validated to ensure the reliability of the values. In addition, the relationship between the type of device or stick and the amount of ROS generated has not yet been studied, as the analysis reported previously involved only one brand of IQOS.

Therefore, we developed a reliable ROS measurement technique in this study to accurately measure the ROS generated from HTPs. We aimed to use the developed method to measure the ROS generated from various HTP devices and sticks to obtain data that would help evaluate the harmfulness of HTP from the perspective of oxidative stress.

## Methods

### Chemicals and reagents

KH_2_PO_4_, K_2_HPO_4_, NaOH, ethanol, H_2_O_2_, and peroxidase from horseradish (HRP) were purchased from Fujifilm Wako Pure Chemical Corporation (Osaka, Japan), and 2′,7′-dichlorodihydrofluorescein diacetate (DCFH-DA) was from Merck (Darmstadt, Germany). Ultrapure water was produced from Milli-Q^®^ IQ 7003 systems (Merck, Darmstadt, Germany). A 25 mM potassium phosphate buffer (KP buffer, pH 7.1) was prepared by mixing 25 mM KH_2_PO_4_ with 25 mM K_2_HPO_4_.

### HTP and reference cigarette

Three different HTP devices were tested in this study: IQOS ILUMA (Philip Moris International, NY, USA), glo hyper+ (British American Tobacco, UK) and Ploom X ADVANCED (Japan Tobacco, Japan). The heat sticks used for each device are listed in Table 1. 3R4F (University of Kentucky, KY, USA) was used as a reference conventional cigarette. HTP heat sticks and conventional cigarettes were used after being placed under 22 °C and 60% humidity conditions for more than 48 h, according to ISO 3402: 1999 [18].

**Table 1:** HTP sticks tested in this study

**HTP device**	**Stick name**	**Taste categories**	**Abbreviation**
IQOS ILUMA	TEREA REGULAR	Regular	TR
	TEREA RICH REGULAR	Regular	TRR
	TEREA MENTHOL	Menthol	TM
	TEREA BLACK MENTHOL	Menthol	TBM
	TEREA PURPLE MENTHOL	Flavored menthol	TPM
	TEREA SUN PEARL	Flavored menthol	TSP

glo hyper+	KENT TRUE TOBACCO	Regular	KTT
	neo Terracotta Tobacco	Regular	NTT
	KENT TRUE MENTHOL	Menthol	KTM
	neo FREEZE MENTHOL	Menthol	NFM
	neo Brilliant Berry	Flavored menthol	NBB
	LUCKY STRIKE TOROPICAL BOOST	Flavored menthol	LTB

Ploom X ADVANCED	MEVIUS RICH	Regular	MR
	CAMEL RICH	Regular	CR
	MEVIUS SHARP COLD MENTHOL	Menthol	MSC
	CAMEL MENTHOL COLD	Menthol	CMC
	CAMEL MENTHOL PURPLE	Flavored menthol	CMP
	CAMEL MENTHOL YELLOW	Flavored menthol	CMY

### Collection of mainstream smoke generated from HTP or cigarette smoke

To align the sample collection conditions, mainstream smoke was generated from all test products following the Health Canada Intense (HCI) regime, which was considered to be similar to actual puffing tomography of smokers [17–19]. As per the HCI regime, mainstream smoke constituents were collected at a puff volume of 55 mL and puff duration of 2 s with 100% blocking of the filter ventilation holes under a room temperature of 22 ± 2 °C and humidity of 60 ± 5%. An LM4E smoking machine (Heinrich Borgwaldt GmbH, Hamburg, Germany) was used to collect the HTPs smoke. For HTPs, the maximum usage time per stick differed for each device: IQOS ILUMA; 6 min, glo hyper+ standard mode; 4 min, glo hyper+ boost mode; 3 min, Ploom X ADVANCED; 5 min. To standardize the puff number for each HTP to 12, the puff intervals for IQOS ILUMA, glo hyper+ standard mode, glo hyper+ boost mode, and Ploom X ADVANCED were set to 30, 21, 16, and 27 s, respectively. Conventional cigarettes were smoked using an LX20 smoking machine (Heinrich Borgwaldt GmbH). The particle phase (PP) of the mainstream smoke was collected on a Cambridge filter pad (44 mm glass fiber filter), and the gas-vapor phase (GVP) remaining after passing through the filter was immobilized into an impinger containing 30 mL of KP buffer. Each PP and GVP sample contained HTP mainstream smoke from three sticks or 3R4F cigarette mainstream smoke from one cigarette.

### Measurement of ROS via chemical assay

ROS levels in the smoke from HTPs and cigarettes were determined using a DCFH fluorescence assay based on a previous method with some modifications [20]. The activated DCFH solution was prepared by dissolving 125 µM DCFH-DA in ethanol (10 mL) deacetylated with 40 mL of 10 mM NaOH for 30 min in dark. The DCFH-HRP solution (containing 5 µM DCFH, 0.5 U/mL HRP) was then prepared by mixing 50 mL of DCFH solution, 200 mL of KP buffer, and 1 mL of 125 U/mL HRP. Afterward, 4 mL of the DCFH-HRP solution was added to 1 mL of a suitably diluted smoke sample extract and incubated for 30 min at room temperature. Thereafter, the fluorescence intensity derived 2′,7′-dichlorofluorescein (excitation wavelength: 495 nm, emission wavelength: 525 nm) was measured using an F-2500 fluorescence spectrophotometer (HITACHI, Tokyo, Japan). The ROS concentration in the sample was expressed as equivalent H_2_O_2_ concentration.

### The limit of detection (LOD) and lower limit of quantitation (LLOQ)

The LOD and LLOQ of the measurement were defined as three times and ten times the standard deviation (SD) obtained from five replicate assays of 10 µM H_2_O_2_ standard solution, respectively.

### Calibration curve

H_2_O_2_ standard reagents (10, 20, 50, 100, 200, 300, 400, 500 and 600 nM H_2_O_2_) were used to create calibration curves. Two calibration curves with different range (10–200 and 200–600 nM) were prepared, and a weighting factor of 1/x was used.

### Precision and accuracy

The assay precision was evaluated based on the relative standard deviations (RSDs, %). HTP mainstream smoke PP or GVP intact sample and sample spiked with various concentrations of H_2_O_2_ (20 or 100 nmol/stick for PP and 2 or 10 nmol/stick for GVP) were measured five times per day, and the RSDs of the measured values were then calculated. The analytical recovery rates (ratio of intact sample concentration to spiked sample, %) were used to evaluate the assay accuracy.

### Statistical analysis

Statistical analysis was performed using the open-source software R. Student’s *t*-test was used to compare the means of two groups. Tukey’s test was used to compare the means of three or more groups. Statistical significance was set at *p* < 0.05.

## Results

### LOD, LLOQ and calibration curves for DCFH fluorescence assay

**Fig. 1:**
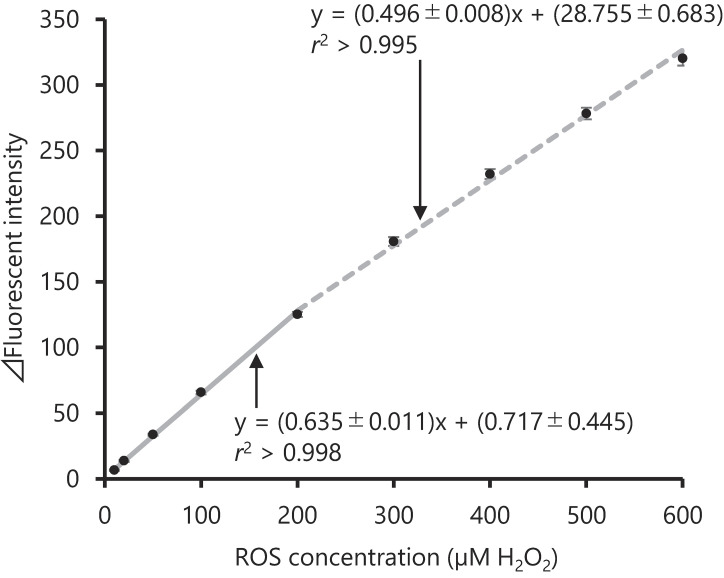
Calibration curve of H_2_O_2_ standard Two calibration curves with ranges of 10–200 and 200–600 nM were prepared. The regression equations were obtained from five independent assays. Mean ± SD values are given for the slope and y-intercept. ROS: reactive oxygen species, SD: standard deviation

First, LOD and LLOQ were examined to determine the lowest ROS concentration that could be quantified, as shown in the Method section. The LOD was 1.35 nM and the LLOQ was 4.50 nM. The regression equations for the calibration curves in the ranges of 10–200 nM and 200–600 nM obtained from five independent assays were y = (0.635 ± 0.011)x + (0.717 ± 0.445) and y = (0.496 ± 0.008)x + (28.755 ± 0.683), respectively (Fig. 1). Both calibrations achieved *r*^2^ > 0.995, and the errors in the inverse estimates were less than 10% over the entire range, indicating that ROS could be measured at the 10–600 nM range.

### Precision and accuracy

To confirm the precision and accuracy of the ROS measurement in an actual HTPs smoke, five samples of heated tobacco PP or GVP, each spiked with a fixed volume of H_2_O_2_ standard solution, were prepared and assayed. The RSDs of the five samples were lower than 3.10% and 2.06% for the PP and GVP samples, respectively, confirming the high analytical precision of the assay (Table 2). When H_2_O_2_ was added to the PP and GVP samples, the analytical recoveries were 98.6–103.1% and 91.2–106.1%, respectively, proving that the assay is sufficiently accurate (Table 2). These results confirm that the assay can accurately measure ROS levels in HTP mainstream smoke.

**Table 2:** Assay precosion and accuracy

**Sample**	**Spiked** **concentration** **(nmol H_2_O_2_/stick)**	**ROS amount** **(nmol H_2_O_2_/stick,** **Mean ± SD)**	**RSD** **(%)**	**Analytical recovery** **(%, Mean ± SD)**
PP	0	28.6 ± 0.333	1.16	-
	20	48.3 ± 1.50	3.10	98.6 ± 7.49
	100	132 ± 1.80	1.37	103.1 ± 1.80

GVP	0	6.81 ± 0.119	1.75	-
	2	8.63 ± 0.161	1.87	91.2 ± 8.06
	10	17.4 ± 0.359	2.06	106.1 ± 3.59

### Measurement of ROS in various HTPs mainstream smoke

**Fig. 2:**
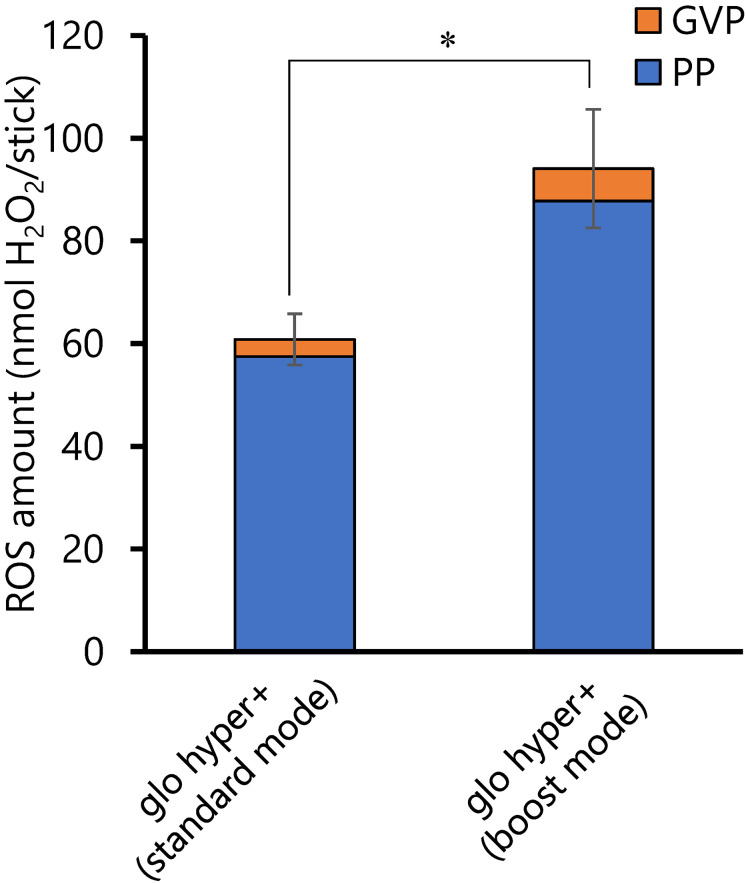
Comparison of the amounts of ROS from glo hyper+ in different heating modes Amounts of ROS from glo hyper+ with KTT sticks in the standard and boost modes were determined. The PP of the mainstream smoke was collected on a Cambridge filter pad (44 mm glass fiber filter), and the GVP remaining after passing through the filter was immobilized into an impinger containing 30 mL of KP buffer. Data are shown as the mean value ± SD (*n* = 5). *p < 0.01. ROS: reactive oxygen species; KTT: KENT TRUE TOBACCO (regular taste stick for glo hyper+); PP: particle phase; GVP: gas-vapor phase; KP buffer: 25 mM potassium phosphate buffer; SD: standard deviation

**Fig. 3:**
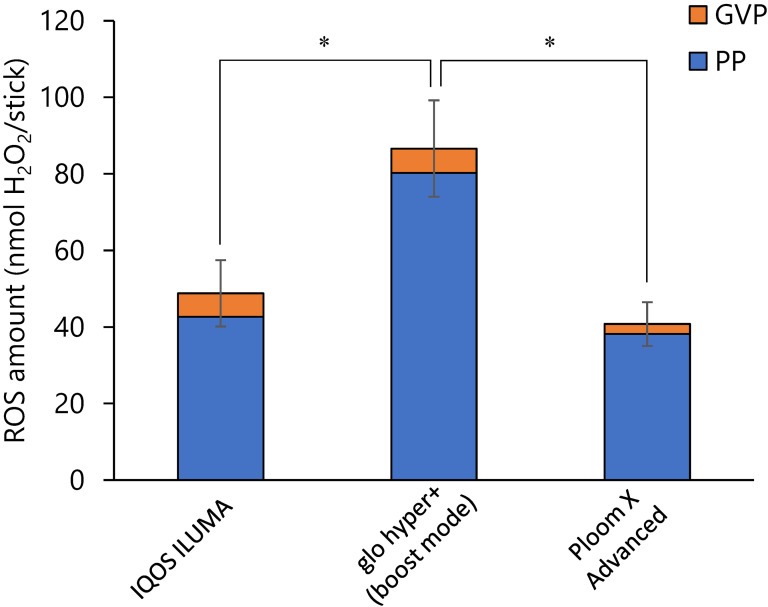
Comparison of the ROS generated from three HTP devices Amounts of ROS from IQOS ILUMA, glo hyper+ and Ploom X ADVANCED were determined. The PP of the mainstream smoke was collected on a Cambridge filter pad (44 mm glass fiber filter), and the GVP remaining after passing through the filter was immobilized into an impinger containing 30 mL of KP buffer. Data are presented as the mean value ± SD for each device’s six different sticks shown in Table 1. *p < 0.01. ROS: reactive oxygen species; HTP: heated tobacco product; PP: particle phase; GVP: gas-vapor phase; KP buffer: 25 mM potassium phosphate buffer; SD: standard deviation

The amount of ROS in mainstream smoke was measured during smoking with each of the six different sticks in three different HTP devices: IQOS ILUMA, glo hyper+, and Ploom X ADVANCED, according to established methods. We tested two sticks of each type: regular, menthol and flavored menthol. The amount of ROS in the mainstream smoke from conventional cigarettes was also compared. The results are shown in Table 3 and are calculated as the amount of ROS per stick. The results showed that the PP and GVP of all HTP brands contained ROS (33.7–108.4 nmol H_2_O_2_/sticks) but not as much as that of conventional cigarettes (2672 nmol H_2_O_2_/sticks). In addition, the percentage of PP in the total mainstream ROS content was greater than 80% in all cases, indicating that PP contained more ROS than GVP. To clarify the relationship between smoking temperature and ROS production, we compared the standard and boost modes of glo hyper+. According to the manufacturer’s information, the heating temperatures in standard and boost modes are 250 °C and 270 °C, respectively. The amount of ROS generated was significantly higher in the boost mode by a factor of approximately 1.5 (Fig. 2). Results of the comparison of ROS generation for each device showed that the glo hyper+ generated approximately twice as much ROS as the IQOS ILUMA and Ploom X ADVANCED (Fig. 3). No differences in ROS generation were observed based on taste category (regular, menthol, or flavored menthol).

**Table 3:** ROS contents in PP and GVP of mainstream smoke

**Device**	**Stick**	**ROS amount (nmol H_2_O_2_/stick)**		

		**PP**	**GVP**	**Total**
		
		**Mean ± SD**	**Mean ± SD**	**Mean ± SD**
IQOS ILUMA	TR	44.3 ± 3.1	7.8 ± 1.4	52.0 ± 3.9
	TRR	45.3 ± 9.5	5.7 ± 0.6	51.0 ± 9.6
	TM	26.4 ± 3.2	7.3 ± 0.5	33.7 ± 2.9
	TBM	45.9 ± 4.8	5.6 ± 0.5	51.5 ± 5.0
	TPM	40.0 ± 0.8	5.2 ± 0.4	45.3 ± 0.8
	TSP	53.8 ± 3.1	5.4 ± 0.2	59.3 ± 3.0

glo hyper+ (standard mode)	KTT	57.4 ± 4.8	3.4 ± 0.3	60.9 ± 5.0

glo hyper+ (boost mode)	KTT	87.8 ± 11.7	6.4 ± 0.6	94.1 ± 11.5
	NTT	76.1 ± 6.0	4.8 ± 0.4	80.8 ± 6.4
	KTM	68.6 ± 8.8	7.7 ± 0.5	76.3 ± 8.7
	NFM	77.7 ± 7.4	6.4 ± 0.5	84.1 ± 7.9
	NBB	101.8 ± 10.1	6.7 ± 0.9	108.4 ± 9.3
	LTB	69.5 ± 5.6	6.4 ± 0.4	75.9 ± 5.6

Ploom X ADVANCED	MR	48.1 ± 9.6	2.3 ± 0.2	50.4 ± 9.5
	CR	31.0 ± 7.7	2.7 ± 0.6	33.7 ± 7.8
	MSC	39.8 ± 3.8	3.1 ± 0.2	42.8 ± 3.8
	CMC	34.8 ± 2.3	2.1 ± 0.3	36.8 ± 2.3
	CMP	36.7 ± 6.5	2.6 ± 0.1	39.4 ± 6.4
	CMY	38.7 ± 10.5	2.7 ± 0.2	41.4 ± 10.3

(Combustible cigarette)	3R4F	2564 ± 138	107 ± 26	2672 ± 137

## Discussion

The generation of ROS from conventional cigarettes has long been recognized and considered a factor that can harm the health of active and passive smokers [9]. However, studies on the amount of ROS generated from HTPs are limited, and the actual situation remains unclear. In this study, the ROS generated from various HTP devices were measured, and it was found that all the devices generated varying amounts of ROS.

In previous methods for measurement of ROS generated from tobacco products using DCFH, ROS in the PP reacted with DCFH at the same time as it was extracted from the filter, and that in the GVP reacted with DCFH during collection [14, 16, 20]. However, our preliminary investigation confirmed that the method of simultaneous collection, extraction, and reaction with DCFH resulted in a high concentration of ROS that exceeded the quantifiable range (600 nM). Therefore, one of the reasons why the measured ROS values differed from those in the previous study could be that these methods were modified to collect mainstream smoke or extract it from the filter and react with DCFH in separate processes. The values obtained in this study can be considered reliable, because we thoroughly examined the calibration curve (Fig. 1), precision, and accuracy (Table 2) using an H_2_O_2_ standard solution and heated tobacco mainstream smoke extracts. This method could be applied to a wide variety of samples where ROS is expected to be generated, including e-cigarettes, water pipes, and other aerosol-based products.

The amount of ROS generated by the HTPs varied approximately 3-fold depending on the devices and the bland of sticks. Results of device-by-device comparison showed that IQOS ILUMA and Ploom X ADVANCED showed similar ROS generation, whereas only glo hyper+ showed a tendency to generate higher ROS. The detailed cause of this difference in ROS generation is unknown. The differences in the heating system and stick structure of each device may have caused the amount of ROS generated to vary even when the smoking was based on the same HCI regime. In addition, ROS generation increased at higher temperatures for glo hyper+, suggesting that the heating temperature may be one of the factors that influenced ROS generation (Fig. 2). A strong positive correlation between heating temperature and ROS production has been reported for electronic cigarettes: the higher the power per unit area, the higher the ROS production [20].

The present study confirmed that 33.7–108 nmol H_2_O_2_/stick of ROS was generated from HTPs, which seems small compared to the amount of ROS generated from conventional cigarettes. However, based on the other literature and assumption of a respiration rate of 17.3 m^3^/day, the daily intake of ROS in the air is calculated to be approximately 22–300 nmol/day [21, 22]. Hence, if one pack (20 sticks) of HTPs is smoked daily, the daily ROS intake would be 674–2160 nmol/day, which is 2.2–96 times the amount of ROS exposure in daily life. Therefore, HTPs users are likely to ingest ROS amounts far beyond their daily routines. Regarding particles and gases, other reports have shown that particles easily reach the alveoli and gases are easily absorbed by the mucosa of the upper respiratory tract [23, 24]. Based on the results of this study, it is likely that the majority of the ROS generated from HTPs are contained in PP, indicating that the effects of ROS on the lungs are significant.

The threshold for ROS inhalation exposure remains unclear, which could result in adverse health effects. However, Ernstgard et al. reported that inhalation exposure to 2.2 ppm of H_2_O_2_ can cause respiratory effects [25]. The ROS concentrations in the HTPs smoke calculated in this study ranged from 1.23 to 3.93 ppm. This assay measures not only H_2_O_2_ but also a combination of various ROS, such as organic peroxides and other ROS. Therefore, there is a high possibility that the ROS from HTPs may cause health hazards.

We previously confirmed in a cell-based study that HTPs smoke causes oxidative stress [26]. A study on mice also showed that HTPs smoke causes oxidative stress in lung tissues [27]. We speculate that the ROS detected in this study may have contributed to some of these oxidative stresses. However, the ROS levels measured in this study were limited to those that could be measured using DCFH, and furthermore, this method was unable to distinguish between different ROS species. As for other ROS, free radicals have been detected in the gas phase of HTPs using combined analysis of spin-trapping agents and electron paramagnetic resonance [28]. Cigarette smoke also contains components such as unsaturated carbonyls, benzosemiquinones, benzo[*a*]pyrene, and metal ions that generate ROS via reactions with biological components owing to their redox activity [29–31]. In addition, the stimulation of cigarette smoke may alter the amount of ROS generated endogenously by the mitochondrial electron transfer system, NADPH oxidase, xanthine oxidase, and carbon monoxide synthase. To determine oxidative stress caused by tobacco products, it is necessary to consider the amount of various chemicals related oxidative stress, rather than amounts of ROS alone. This is because a complex combination of these factors determines the degree of oxidative stress to the organism. Nevertheless, one of the important findings of this study is that we were able to clarify that HTPs produce ROS amounts that may affect human health, even considering only the ROS that could be detected by the DCFH-based method.

One limitation of this study is the insufficient consideration of the puffing topography of HTPs users. While the study employed the HCI method, which closely mimics the puffing topography of conventional cigarette smokers, differences in puff parameters—such as volume, duration, and number—between HTP users and the HCI method may exist. Therefore, ROS exposure should be assessed cautiously, incorporating results from puffing topography studies specific to each device user.

## Conclusion

In this study, the ROS generated from HTPs were measured via a chemical assay using DCFH. Three different devices and six different sticks of each device were tested, and it was found that ROS were generated from all HTPs. Results also showed that users of HTPs were exposed to more ROS than those in normal life. Furthermore, the concentration obtained in this study are expected to adversely affect the human respiratory tract. The results of this study provide valuable data that can serve as a basis for evaluating the toxicity of HTPs, especially oxidative stress-related toxicity.

## Abbreviations

HTPs: heated tobacco products
ROS: reactive oxygen species
DCFH: 2′,7′-dichlorodihydrofluorescein
HRP: peroxidase from horseradish
DCFH-DA: 2′,7′-dichlorodihydrofluorescein diacetate
KP buffer: 25 mM potassium phosphate buffer
HCI: Health Canada Intense
PP: particle phase
GVP: gas-vapor phase
LOD: limit of detection
LLOQ: lower limit of quantitation
SD: standard deviation
RSD: relative standard deviation

## Declarations

### Availability of data and material

Raw data used in this study are available on request from the corresponding author.
